# Cancer Cell Colonisation in the Bone Microenvironment

**DOI:** 10.3390/ijms17101674

**Published:** 2016-10-03

**Authors:** Casina Kan, Geoffrey Vargas, François Le Pape, Philippe Clézardin

**Affiliations:** 1 National Institute of Health and Medical Research (INSERM), UMR 1033, Lyon 69372, France; casina.kan@inserm.fr (C.K.); geoffrey.vargas@inserm.fr (G.V.); francois.le-pape@inserm.fr (F.L.P.); 2 Faculty of Medicine RTH Laennec, University of Lyon, Villeurbanne 69372, France

**Keywords:** bone, metastasis, cancer, microenvironment, metastatic niche

## Abstract

Bone metastases are a common complication of epithelial cancers, of which breast, prostate and lung carcinomas are the most common. The establishment of cancer cells to distant sites such as the bone microenvironment requires multiple steps. Tumour cells can acquire properties to allow epithelial-to-mesenchymal transition, extravasation and migration. Within the bone metastatic niche, disseminated tumour cells may enter a dormancy stage or proliferate to adapt and survive, interacting with bone cells such as hematopoietic stem cells, osteoblasts and osteoclasts. Cross-talk with the bone may alter tumour cell properties and, conversely, tumour cells may also acquire characteristics of the surrounding microenvironment, in a process known as osteomimicry. Alternatively, these cells may also express osteomimetic genes that allow cell survival or favour seeding to the bone marrow. The seeding of tumour cells in the bone disrupts bone-forming and bone-resorbing activities, which can lead to macrometastasis in bone. At present, bone macrometastases are incurable with only palliative treatment available. A better understanding of how these processes influence the early onset of bone metastasis may give insight into potential therapies. This review will focus on the early steps of bone colonisation, once disseminated tumour cells enter the bone marrow.

## 1. Introduction

Bone metastases are a frequent complication of solid cancers [1]. The establishment of bone metastasis is a considerable cause of morbidity, often resulting in bone pain, spinal cord compression, hypercalcemia and pathological fractures, ultimately resulting in the need for surgery [2]. Different tumours have varying levels of propensity to metastasise to the bone. Solid epithelial cancers are prone to develop bone metastasis, notably breast and prostate cancer, but also to a lesser extent lung, kidney and melanoma. Bone metastases are observed to affect 65%–75% of advanced breast and prostate cancer patients [3]. These cells have a particular affinity for bone: this may be due to the expression of genes that predispose them to home to the bone marrow, although it is also possible that these cells acquire osteomimicry after localisation within the bone compartment. Bone metastases are predominantly osteoblastic in prostate cancer, and a mixture of osteolytic and osteoblastic in breast cancer [2].

The establishment of cancer cells in the bone marrow requires multiple steps, whereby cells need to leave the primary tumour and then adapt and survive in a physiologically different environment. The local microenvironment, or premetastatic niche, may be modified through the secretion of factors by cancer cells to establish favourable conditions for metastasis. For instance, cancer cell secretion of lysyl oxydase (LOX) is able to increase extracellular rigidity by reticulation of collagen and thus promote cancer cell anchorage [4]. In order to extravasate and survive in the circulation, tumour cells commonly undergo epithelial to mesenchymal transition (EMT), which allows cells to adopt a mesenchymal-like phenotype. These steps are essential for tumour cells to seed to distant sites such as bone [5,6]. This process plays a pivotal role in the initial steps of the metastatic cascade (reviewed in [7]). EMT is defined by the loss of epithelial markers (claudin, cytokeratin, and E-cadherin) and the gain of mesenchymal markers (N-cadherin, vimentin, fibronectin, and smooth muscle actin). Tumour cells that have begun the process of EMT lose expression of molecules responsible for cell-cell junctions such as E-Cadherin and β-catenin by the action of well-described EMT-actors such as Snai1, Twist, Slug and Zeb1/2 transcription factors. In parallel, tumour cells acquire the capacity to be motile by expressing N-Cadherin and vimentin, which are responsible for cytoskeleton rearrangement and lamellipodia formation. The ability for cells to undergo EMT is thought to be related to the ability to self-renew and differentiate into different tumour cell types, also known as “stemness” and adaptability, leading to resistance to chemotherapy [7,8]. It is generally acknowledged that disseminated tumour cells (DTCs) are required to undergo EMT-reversal. This process is known as mesenchymal-to-epithelial transition (MET) whereby cells regain their epithelial phenotype to seed to the metastatic niche, allowing adhesion and anchorage independent growth [9,10]. Hepatocyte-growth factor (HGF) stimulated Twist1 activity, which was shown to positively regulate the MET phenotype to promote breast cancer cell metastasis to bone [10].

Tumour cells that settle in the bone marrow enter a dormant state in specific niches and/or adapt to the bone microenvironment (osteomimicry). Disseminated tumour cells (DTCs) may become active years later as they proliferate and alter the functions of bone-resorbing (osteoclasts) and bone-forming (osteoblasts) cells, disrupting physiological bone remodelling and promoting skeletal destruction. In turn, the release of bone-derived growth factors (transforming growth factor β (TGFβ) or insulin-like growth factor 1 (IGF-1)) and calcium (Ca^2+^) from resorbed bone promote tumour growth [11,12].

This review will focus on the molecules involved in tumour cell dissemination, its homing within the metastatic niches, the establishment of micrometastases and regulation of tumour cell dormancy. Finally, secreted factors which influence bone destruction or tumour cell seeding in the bone microenvironment will also be discussed.

## 2. Osteotropism

### 2.1. Cancer Cell Migration to the Bone

**Figure 1:**
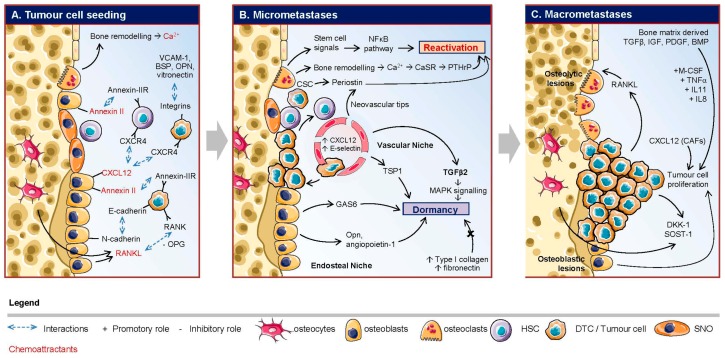
Stages of cancer cell colonisation of the bone. (**A**) Tumour cells are attracted to the high levels of chemoattractants in the bone marrow (red), such as Ca^2+^, CXCL12 (C–X–C motif chemokine ligand 12) and RANKL. In the endosteal niche, hematopoietic stem cells (HSC) and disseminated tumour cells (DTCs) competitively bind to osteoblasts through interactions between CXCR4/CXCL12 and Annexin II/Annexin II receptor. In addition, expression of E-cadherin on the DTC surface can form adherin junctions with N-cadherin expressing osteoblasts as well as form interactions between RANK and RANKL, the latter of which is secreted by osteoblasts and osteocytes. Furthermore, DTCs also express integrins which can interact with a number of factors in the bone marrow (VCAM-1, bone sialoprotein (BSP), osteopontin (OPN), victronectin); (**B**) Once in the bone marrow, DTCs compete with HSCs for the endosteal niche. DTCs are able to proliferate; although a majority of cells die or remain dormant. The local environment has a significant impact on the fate of these DTCs. Local levels of type I collagen and fibronectin have been demonstrated to repress dormancy. Dormant cells have been reported to be found in the vascular niche, in close proximity to capillaries and within regions rich in CXCL12 and E-selection. The vascular niche is able to secrete thrombospondin-1 (TSP1) and Notch-1, which are important for maintaining dormancy. Several studies also show TGFβ2, can play an important role in the maintenance of dormancy through MAPK signalling, while TGFβ1 secreted from neovascular tips is associated with tumour cell reactivation. Periostin can also be secreted by neovascular tips as well as Cancer Stem Cells (CSCs), leading to DTC reactivation. The endosteal (osteoblastic) niche can also maintain tumour cell dormancy through the secretion of GAS6 by osteoblasts and OPN and angiopoietin-1 by spindle-shaped N-cadherin+ osteoblasts (SNOs). To exit dormancy, osteoclasts are able to release stem cell signals, triggering the NFκB pathway. We also know that the release of Ca^2+^ from normal bone remodelling can bind to calcium sensing receptor (CaSR) to stimulate PTHrP, leading to tumour cell reactivation; (**C**) After DTCs are reactivated, they become proliferative and establish macrometastases. Bone matrix derived TGFβ, IGF, PDGF and BMP can promote tumour cell proliferation, as well as Cancer Associated Fibroblasts (CAFs) secreted CXCL12. Once macrometastases are established, the tumour can release factors that drive osteoclasts (M-CSF, TNFα, IL-11 and IL-8) to induce osteolytic lesions through the stimulation of RANKL. Tumour cells have also been shown to release miRNAs that stimulate osteogenesis through the down-regulation of DKK-1 and SOST-1. Finally, association of tumour cells with the endosteal niche has been associated with tumour cell growth.

For cancer cells to migrate from the primary tumour to the bone, the migration of cancer cells may be triggered by chemotaxis in response to different stimuli. The mechanisms by which the breast carcinoma cells migrate and colonise the bone marrow are still only partially understood. While Paget has developed the hypothesis of the “seed and soil” to explain tumour cell tropism to a specific organ [13], it is now clearly recognised that there is a genetic determinant in tropism and the bone colonisation process. Massagué et al. has demonstrated a “molecular signature” acquired by cancerous cells within the primary tumour, allowing them to spread and colonise the bone marrow [14]. This molecular signature includes genes originally involved in bone physiology and these genes are mirrored in the tropism of tumour cells to the bone. Indeed, tumour cells use the same mechanisms employed by hematopoietic stem cells (HSC) and leucocytes to migrate to the bone. This process involves receptors expressed by tumour cells and ligands expressed by the bone microenvironment (Figure 1A).

The main molecules involved in HSC and tumour cell migration are couple chemokine (C–X–C) receptor type 4 (CXCR4) and 6 (CXCR6) and their respective ligands, C–X–C motif chemokine ligand 12 (CXCL12) and 16 (CXCL16). In healthy breast and prostate epithelial cells, CXCR4 and CXCR6 are not expressed, but their expression levels are significantly increased in invasive cancer cells [15,16]. In the bone marrow, osteoblasts constitutively express CXCL12, which can act in unison with sphingosine-1-phosphate (S1P) as a chemo-attractant to regulate HSC homing [15,17]. For HSC, activation of CXCR4 by CXCL12 triggers migration to the bone marrow. In the same manner, overexpression of CXCR4 in breast cancer cell lines increased bone metastasis formation in animal models [14]. Inhibition or over-expression of CXCR4 modifies cancer cell propensity to colonise bone marrow [18,19]. Similarly, inhibition of CXCR4 by pharmacological agents or neutralising antibodies decreases metastatic cancer cell dissemination to the lung and bone [19,20]. CXCL16 is expressed in vivo by bone tissue, including osteocytes [21]. It acts as an in vitro chemotactic agent to promote the migration of CXCR6-expressing prostate cancer cells (PC3 cell line) and therefore promote the formation of bone metastases [21].

High extracellular Ca^2+^ concentration as a result of normal bone remodelling has been demonstrated to act as a chemoattractant for breast cancer cells in vitro [22]. This was shown to be due to Ca^2+^ acting on calcium sensing receptor (CaSR), in line with the role of CaSR in the localisation of HSCs to the bone marrow due to high local Ca^2+^ levels (Figure 1A) [23]. This relates to the “vicious cycle”, which was described by Mundy et al., whereby tumour cells interact with the bone microenvironment to drive disease progression [24]. Under this system, osteoclast-mediated bone resorption occurs when Ca^2+^ binds to CaSR which can in turn stimulate PTHrP in cancer cells in a feed-forward loop [11]. A recent study in breast cancer showed that CaSR stimulates intracrine PTHrP signalling, promoting tumour cell proliferation and survival [12].

In addition, the receptor activator of NFκB ligand (RANKL) is produced by osteoblast in bone and the RANK/RANKL pathway has a key role in osteoclast differentiation [25]. RANK is expressed in several breast and prostate cancer cell lines as well as primary human breast tumours and has been demonstrated to be an important driver of cancer cell migration to the bone [26,27]. RANKL is also a mediator of osteoclast function and survival and is negatively regulated by a decoy receptor, osteoprotegerin (OPG) [25,28]. Direct contact between breast cancer cells and osteoblastic cells induced RANKL and OPG expression by cancer cells [29]. Jones et al. showed RANK-expressing breast and prostate cancer cell lines treated with recombinant RANKL stimulated migration, which was blocked by the decoy receptor OPG [27]. Importantly, osteocytes are a major source of RANKL in adulthood [30,31,32], and there is some evidence in the literature that osteocytes contribute to bone metastasis formation by favouring cancer cell colonisation in the bone marrow [31,32]. Osteoblasts also express Annexin II, which has been shown to promote tumour cell tropism to bone. This has been demonstrated in Annexin II receptor-expressing prostate cancer cells which migrate toward Annexin II [33].

### 2.2. Cell Adhesion

An important part in the mestastatic cascade, loss of cell-adhesion is firstly demonstrated to allow cells to dissociate from the surrounding matrix in order to extravasate into the circulation [34]. After the cell enters the bone marrow, the ability for cells to regain adhesiveness is essential for cell-anchorage. Tumour cells arrive in a complex environment and because they express the same surface molecules as HSCs, tumour cells can be anchored in the bone marrow (Figure 1A). In the bone marrow, HSCs are housed in anatomical entities called “niches”. Each of these niches regulates anchorage, quiescence and proliferation, which will be described in the following section. Several molecules are also known to participate in enhanced adhesion in bone, summarised in Table 1. This includes adherin-junctions within the osteogenic niche, formed by the association of mesenchymal N-cadherin and breast cancer cells which express E-cadherin [35]. Integrins are also important mediators of anchorage of HSCs, since they bind to several extracellular matrix factors and have been shown to be involved in adhesion, migration and invasion to the bone. Integrins αvβ3 and αvβ5 are capable of binding to important bone matrix proteins, bone sialoprotein (BSP), osteopontin (OPN) and vitronectin [36,37]. Integrin αvβ3 is expressed by breast and prostate cancer cells, and overexpressed in an osteotropic model of breast cancer derived from MDA-MB-231 cells, called B02 [38]. Overexpression of integrin αvβ3 increased bone metastasis formation; treatment with non-peptide antagonists blocked bone colonisation in integrin αvβ3-overexpressing cancer cells [38]. Moreover, 66C14 murine cancer cells which specifically metastasise to the lung exhibited bone tropism after overexpression of integrin αvβ3 [39]. Integrin α4β1 (VLA-4) is also expressed by tumour cells and allows their adhesion through its interaction with vascular cell adhesion molecule-1 (VCAM-1) expressed by stromal cells in the bone marrow [40]. Further, osteoblasts and prostate cancer cells express annexin-II and its receptors, respectively, and the adhesion of prostate cancer to osteoblasts, as well as the homing of prostate cancer cells to bone in vivo, are limited by annexin II antibody [33].

## 3. Disseminated Tumour Cells (DTCs) in the Bone Marrow

In a large-scale study of early-stage breast cancer patients, DTCs were identified in 30% of bone marrow aspirates at time of diagnosis [53]. Similarly, DTCs were identified in the bone marrow for 90% of advanced-stage prostate cancer patients [54,55,56]. The processes that determine the fate of DTCs in the bone marrow remain unclear; however, the presence of DTCs is reported to be a negative prognostic indicator in breast and prostate cancer [53,57,58,59,60]. The influence of bone marrow niches on DTCs and the molecules that maintain dormancy will be discussed in the following sections.

### Bone Marrow Niches

Endosteal and vascular niches are crucial regulators of normal and malignant stem-cell behaviour in the bone marrow. Quiescent HSCs are enriched in the endosteal niche lining the bone surface, whereas the vascular niche is oxygenated and stimulates proliferation and differentiation of HSCs [61]. The adhesion molecule E-selectin which is selectively expressed by endothelial cells in the vascular niche promotes HSC proliferation [44]. Further, expansion of the vascular niche for HSCs has been demonstrated to be stimulated by Notch signalling in endothelial cells [62]. The proximity of HSCs to the endosteal niche is responsible for osteoblast-driven maintenance and quiescence of HSCs [61]. Specifically, this quiescence of HSCs seems to be dependent on the CXCL12/CXCR4 axis, as treatment with small molecule CXCR4 antagonist AMD3100 rapidly mobilised HSCs from the bone marrow [63]. Additional mechanisms are likely to be involved in maintaining HSC quiescence. For instance, growth arrest-specific 6 (GAS6), a receptor for annexin-II, is expressed by osteoblasts and mediates HSC quiescence [45]. Additionally, the endosteal niche is home to a particular spindle-shaped N-cadherin+ osteoblast (SNO) cell population. SNOs regulate quiescence of HSC through interaction with N-cadherin and secretion of angiopoietin and OPN [47].

Breast tumour cells were found to preferentially locate to osteoblast-rich regions following chemical alteration of the endosteal niche with the bisphosphonate zoledronic acid [64]. Similarly, ablation of the endosteal niche by conditional knock-out was shown to be critical for prostate tumour cell growth in bone [65]. It has been shown that epithelial-like cancer cells interact with osteogenic cells and other stromal cells in the bone microenvironment through interactions between E-cadherin and N-cadherin [35]. Furthermore, biphoton analysis of fluorescently labelled cancer cells in the bone marrow suggests the involvement of osteoblast lineage cells and that these cancer cells occupy the same endosteal niche as HSCs [66]. Indeed, DTCs in the bone marrow interact with the endosteal and vascular niches, influencing DTC proliferation and outgrowth. Recently, real-time in vivo microscopy of breast tumour xenografts showed that dormant DTCs were preferentially located in E-selectin-rich vascular regions [67]. The use of highly specific inhibitors of E-selectin inhibited entry of breast cancer cells into the vascular niche, whereas inhibition of the CXCR-4/CXCL-12 axis induced mobilisation of dormant breast cancer cells from the vascular niche into circulation [67]. These results [67] are in contrast with previous findings showing that dormant prostate cancer cells, which express CXCR4, compete with HSCs for endosteal niche support [65,68]. DTCs likely benefit from some additional cues in the endosteal niche. A recent study reported that DTCs from prostate cancer cell lines in the endosteal niche take on characteristics of cancer stem cells (CD133+/CD44+, increased KLF 4, Bmi-1, and Nanog mRNA levels) (Figure 1B), a process which was controlled by GAS6-mediated mTOR signalling [61]. Overall, this concept of niche support for DTCs is still in the early stages of investigation, and warrants further investigation.

## 4. The Fate of DTCs in the Bone Marrow

### 4.1. Tumour Dormancy

Several clinical observations showed that DTCs in the bone marrow do not proliferate immediately but enter a state of dormancy, whereby a group of cancerous cells cannot grow beyond a certain size [69]. It is possible that dormant DTCs never develop cancer or they can exit dormancy to form bone metastasis, potentially many years after diagnosis [70]. Several theories exist to explain how DTCs remain dormant, with the bone microenvironment playing a crucial role in this phenomenon. Tumour dormancy can be defined as when the cell arrests and enters a quiescent state (G0) [71]. Dormancy allows DTCs to adapt to the microenvironment while at the same time they are protected from the immune system and different therapies. The bone microenvironment is composed of different cell types important for the survival and maintenance of HSCs and potentially tumour cells [72] (immune surveillance and survival will be discussed in the next section). Growth-arrested DTCs have been found in close proximity to the perivascular niche [48]. Furthermore, dormant or slow-growing prostate cancer cells have been demonstrated to localise to the endosteal niche [68].

As discussed, the annexin II and its receptor are expressed by osteoblasts and prostate cancer cells, respectively [33]. The binding of prostate cancer cells to annexin II induces the expression of the growth arrest-specific GAS6 receptors Axl, Sky and Mer, which in the hematopoietic system, induces dormancy [45]. In addition, GAS6 produced by osteoblasts prevents prostate cancer cell proliferation and protects prostate cancer cells from chemotherapy-induced apoptosis [45]. Axl, and hypoxia-inducible factor-1α (HIF-1α) were co-expressed in prostate cancer tissue and bone metastases [73]. In hypoxic environments such as tumours and the bone microenvironment, it is suggested that tumour mass is restricted by the lack of sufficient vascularisation [71]. One study showed that stable bone microvasculature maintained a dormant niche by promoting tumour cell quiescence through Notch-1-mediated regulation of neovascular tips and the angiocrine tumour suppressor functions of thrombospondin-1 (TSP-1) [48].

TGFβ2 is a bone marrow derived factor which promotes DTC dormancy through TGFβ-R3 and MAPK p38α/β signalling [51]. In the same study [51], treatment with TGFβ1 switched off dormancy, leading to rapid tumour growth in vivo, suggesting that TGFβ2 alone is a mediator of dormancy. It is possible that TGFβ regulation of dormancy could act through the bone morphogenetic protein (BMP) pathway. This signalling pathway is well characterised in bone formation [74]. Recently, indolent prostate cancer cells have been reported to secrete SPARC, which can induce BMP7 secretion by bone marrow stromal cells [50], further supporting the role of BMP7 in inducing prostate cancer stem cell senescence [49].

### 4.2. Survival

It is generally accepted that a majority of DTCs do not survive, due to the inefficient metastatic process. The bone marrow has been postulated to provide survival signals, whereby the bone marrow may be a protective environment for DTCs in patients undergoing chemotherapy [75,76]. For cancer cells to be established in the bone, they will need to adapt to the new microenvironment to survive and proliferate [77,78,79]. In one study, breast cancer stem cells were demonstrated to modify the metastatic niche through the secretion of stromal periostin [71]. Fibroblast cells adjacent to tumour cells, also known as cancer-associated fibroblasts (CAFs), can be altered by neighbouring tumour cells. In one study, treatment with conditioned medium from the breast cancer cell line SUM102 elevated levels of CXCR4 in CAFs [80]. Furthermore, CAFs have also been reported to secrete high levels of CXCL12 which were demonstrated to enhance the proliferation of breast cancer cells [81]. Intriguingly, CAFs secreted higher levels of CXCL12 than the breast cancer cell line MCF-7, which promoted tumour growth and angiogenesis through the recruitment of endothelial progenitor cells. This suggests that CAFs are able to act independently of tumour cells to further exacerbate the bone metastasis cascade. In this way, it is proposed that CAFs can add selection pressure to heterogeneous tumour cell populations, priming them for bone metastasis through exposure to CXCL12 and IGF1 [43]. “Primed” cancer cells were found to exhibit enhanced Src activity and bone metastatic ability. This is supported by a previous study, whereby intracellular Src enhanced PI3K-AKT signalling through stromal CXCL12, resulting in increased breast cancer metastasis to the bone marrow [42]. Fibroblasts from the bone marrow stroma have also been reported to secrete factors that may play a part in cancer cell metastasis in the bone [82].

Bone turnover may have an important role on bone metastasis [83,84]. In ovariectomised mice, no effect was observed for initial tumour cell numbers in the bone compared to sham operated mice, suggesting that ovariectomy does not affect tumour cell homing, however breast cancer cell colonies were increased [85]. Gene expression analysis showed increased RANKL and DKK-1 and decreased OPG; this was accompanied by increased MMP-9 and cathepsin K activity, suggesting increased bone resorption through elevated osteoclast activity [85]. This was proposed to be due to the stimulation of the mesenchymal stem cell niche and thereby promote DTC proliferation [86]. Similar to the breast cancer model, castration in mouse models of prostate cancer resulted in osteoclastic bone resorption, which increased bone metastasis [87]. This work supports the clinical observation that higher bone turnover is associated with poorer outcome in patients with bone metastasis [88,89,90,91].

In addition, the immune system is also involved in reducing tumour cell proliferation through a process known as “immune-surveillance”, which balances proliferating cells and dying cells [92]. This is the process whereby immune cells detect and/or eliminate tumour cells. Tumour cells have been reported to find refuge in the bone, promoting DTC survival [93]. This has been postulated to be due to a dampened immunity which protects HSCs in the bone compartment [93]. The same mechanisms, which maintained HSC quiescence, are also implicated in tumour cell dormancy (CXCR4, CXCL12 and angiopoietin-1) [46]. To adapt, survive and grow in the bone microenvironment, cancer cells must mimic bone cells (osteomimicry) through the expression of molecules and factors usually expressed by osteoblasts or osteoclasts. Paired immunochemistry on primary breast tumour samples and associated liver, lung or bone metastases showed that only bone metastatic cancer cells express bone proteins such as cathepsin K, osteonectin, cadherin-11, connexin-43 and RUNX2 [94,95]. Moreover, some experiments have shown that cancer cells can fuse with macrophages or induce multinucleated giant cells by fusion with osteoclast precursors, leading to cancer cells with osteoclast properties [96]. Breast cancer cells express a number of osteoclast acting factors (PTHrP, IL-11, IL-6, TNFα, M-CSF) to promote RANKL. The expression of these molecules may confer a survival advantage in immunosurveillance or in the active colonisation of the bone marrow [75].

### 4.3. Reactivation

The latency period observed in metastatic relapse refers to the time for DTCs to adapt and escape the metastatic niche (Figure 1B). Interestingly, the role of the vascular niche in maintaining tumour cell dormancy is not so clear-cut, and that sprouting neovasculature can promote metastatic outgrowth as a result of active TGFβ1 and periostin secreted from endothelial tip cells [48]. Within the metastatic niche, extracellular Ca^2+^ can also activate dormant tumour cells [97]. However, recent evidence in multiple myeloma showed that dormant tumour cell reactivation is dependent on its localisation within the metastatic niche. That is, extrinsic factors from the endosteal niche are able to “switch on” dormant tumour cells [97]. For instance, breast cancer cell expression of VCAM-1 has been shown to bind with integrin α4β1 to promote the recruitment of osteoclast precursors, leading to tumour cell reactivation [52]. Tumour cell development of an osteoclastic niche creates a shift in bone homeostasis, triggering the release of stem cell signals to stimulate metastatic outgrowth, possibly through the NFκB pathway [52]. Therefore, DTC reactivation also depends on intrinsic factors from the tumour cells to permit self-renewal (cancer stem cell-like) in addition to environmental factors from the metastatic niche.

In addition to the metastatic niche, a number of proteins have been reported to be able to reactivate dormant DTCs. For instance, microenvironments rich in type I collagen [97,98] or fibronectin [99] have been associated with diminished dormancy. Cathepsin K, which is secreted by osteoclasts, has been shown to cleave MMP-9 [100] and CXCL12 [101]. Disruption of these proteins may perturb bone homeostasis and result in the mobilisation of hematopoietic progenitors, thereby promoting DTC activation [70,101]. In human breast cancer cells, the expression of inhibitor of differentiation 1 (ID1) and 3 (ID3) has been demonstrated to reinitiate metastatic colonisation through bypassing senescence and promoting extravasation [102]. Further, DTC reactivation may be regulated by TGF-β, which can induce ID1 [103].

## 5. Tumour Outgrowth and Secretion of Factors

Metastatic cancer cells are not solely responsible for the destruction of bone. This process mainly involves osteoblasts and osteoclasts and they are essential for metastatic tumour cell establishment in the bone (Figure 1C). Osteoblast production of RANKL/OPG can also regulate osteoclast formation through PTHrP, interleukins (IL-1, and IL-6) and prostaglandin E2 (PGE2) [24]. Further, osteoclasts were reported to stimulate RANKL on the osteoblast surface in mouse models of breast cancer bone metastasis [104]. Activated TGFβ, IGFs, PDGF and BMP family members released from the bone matrix have also been shown to enhance tumour cell proliferation [105,106].

In addition, osteoclasts can secrete factors that promote tumour growth such as microRNAs (miRNAs), some of which have been shown to modulate bone function [107]. A number of miRNAs have been reported to be differentially expressed following RANKL-induced osteoclastogenesis in murine preosteoclast RAW264.7 cell lines [108,109]. Interestingly, the same study demonstrated that treatment of conditioned media from bone-metastatic cells (4T1.2 and TSU-Pr-1-B2) were found to induce osteoclast differentiation similar to that induced by RANKL [108]. This was further confirmed by mammosphere assays whereby breast cancer cells co-cultured with osteoblast precursors were able to accelerate tumour cell proliferation; this effect was not observed in monocyte co-cultures or after differentiation to osteoclasts [35]. Moreover, exosome-like vesicles secreted from tumour cells were able to significantly impair bone metastasis [110]. This further substantiates our understanding of how secreted factors, including miRNAs, are able to regulate adjacent cells in a paracrine manner.

In addition to miRNAs, tumour cells can release factors that can interfere with normal bone resorption. This includes inhibitors of osteoblast differentiation such as dickkopf-1 (DKK-1) and sclerostin (SOST) [111]. Osteoclast activity has also been demonstrated to be stimulated by M-CSF, TNFα and interleukins (IL-8, and IL-11) from breast cancer cells [111]. Finally, cathepsin K produced by tumour cells has been shown to promote tumour cell invasiveness and contribute to bone degradation [94,112].

## 6. Conclusions

While early detection and conventional treatments are effective for breast cancer management, the development of bone metastasis remains a major cause of death. Patients diagnosed with bone cancer are considered incurable. To date, no effective treatments for bone metastasis exist, only therapies that aim to limit the bone degradation such as denosumab (anti-RANKL monoclonal antibody) and bisphosphonates. In this review, we described the main mechanisms that allow cells to disseminate and colonise in the bone marrow by highlighting the importance of the bone microenvironment. It is clearly recognised that the tumour cells use the same processes as HSCs to migrate and anchor in the bone marrow. Furthermore, the dormancy mechanisms give rise to the formation of bone metastases, sometimes years after the detection of primary cancer. The study of DTCs in the bone marrow can be improved through the implementation of different in vitro and in vivo models of the bone metastatic niche [113]. Throughout this review, we have mentioned that osteocytes are the key producers of RANKL, DKK-1 and SOST-1 in adult bone. As the most abundant bone cell, osteocytes may be interesting drug targets, through the use of novel anti-resorptive and anabolic agents and monoclonal antibodies against osteocyte-associated sclerostin (AMG 785 or romozumab) [114]. These agents are currently under clinical trials for osteoporosis; however, a greater need for research in osteocytes in the area of bone metastasis is desired. A better understanding of these mechanisms could lead to new targets for treatments that could maintain cells in a non-proliferative state as well as prevent cell anchorage in the bone marrow.

## Figures and Tables

**Table 1:** Key proteins for tumour cell regulation in the bone.

Function	Protein	Description	Disease	Reference
Homing	SIP	SIP can act in unison with CXCL12 as a chemoattractant	–	[17]
	CXCR4/CXCL12	CXCR4-expressing cancer cell migration to the bone is mediated by osteoblast derived CXCL12.	Breast cancer Prostate cancer	[19]
	CXCR6/CXCL16	CXCL16 is expressed in bone tissue and promotes migration of CXCR6-expressing cancer cells in vitro.	Prostate cancer	[21]
	Ca^2+^/CaSR	Ca^2+^ from bone remodelling stimulates migration of CaSR-expressing cancer cells.	Breast cancer	[23]
	RANK/RANKL	RANK/RANKL axis promotes cancer cell migration by mediating cytoskeleton rearrangement in vitro.	Breast cancer Prostate cancer	[27]
	Annexin II/Annexin IIR	Annexin II produced by osteoblast and endothelial cells promotes the migration of cells expressing annexin II receptor.	Prostate cancer	[33]
Adhesion	CXCR4/CXCL12	CXCL12 in media from human primary bone-marrow has chemotactic properties. Blocking with neutralising CXCR4 antibodies impaired migration.	Breast cancer	[15]
	Annexin II/Annexin IIR	Annexin II is produced by endothelial and osteoblast cells and promotes adhesion of tumour cells expressing annexin II receptor.	Prostate cancer	[33]
	E-cadherin/N-cadherin	E-cadherin was found to be expressed by cancer cells and form adherin junctions with N-cadherin in osteogenic cells.	Breast cancer	[35]
	Integrin αVβ3 and αVβ5	Tumour cells expressing integrin αVβ3 and/or αVβ5 have the capacity to bind bone extracellular proteins such as fibronectin, vitronectin and osteopontin.	Breast cancer	[37,38]
	Integrin α4β1/VCAM1	Integrin α4β1 expression by myeloma cells allow bone cells to bind through VCAM1 interactions.	Multiple myeloma	[40]
Survival	Periostin	CSCs were shown to modify the metastatic niche through stromal periostin expression.	Breast cancer	[41]
	Src	Src-associated gene signature is linked with late-onset bone metastasis. Src activity has been reported in cancer cells “primed” for metastasis in the bone marrow.	Breast cancer	[42,43]
Dormancy	E-selectin and CXCL12	Vascular regions are rich in E-selectin and CXCL12, which is associated with HSC dormancy.	–	[44]
	Gas6/Axl/Sky/Mer	GAS6 is secreted by osteoblasts and is involved in maintaining HSC quiescence.	Prostate cancer	[45]
	Angiopoietin-1	Involved in forming a quiescent niche for HSCs.	–	[46,47]
	TSP-1	The secretion of TSP-1 from endothelial cells induces cancer cell dormancy.	Breast cancer	[48]
	TGFβ 2/BMP7/SPARC	Indolent prostate cancer cells secrete SPARC, which can promote BMP7-mediated senescence.	Prostate Cancer	[49,50]
	BMP7	BMP7 is secreted from bone stromal cells and induces senescence in prostate cancer stem-like cells.	Prostate cancer	[49]
Reactivation	TGFβ1	Secreted TGFβ1 enhances tumour cell formation.	–	[51]
	Integrin α4β1/VCAM1	VCAM1-expressing cancer cells recruit integrin α4β1+ osteoclast progenitors and initiate reactivation through the vicious cycle.	Breast cancer	[52]

## Abbreviations

BMP-7	Bone morphogenic protein 7
CAF	Cancer associated fibroblasts
CaSR	Calcium sensing receptor
CSC	Cancer Stem Cell
CXCL12	C–X–C motif chemokine ligand 12
CXCR4	Couple chemokine (C–X–C) receptor type 4
DTC	Disseminated tumour cell
EMT	Epithelial-to-mesenchymal transition
GAS6	Growth arrest-specific 6
HSC	Hematopoietic stem cell
ID1 or 3	Inhibitor of differentiation 1 or 3
IGF1	Insulin-like growth factor 1
NFκB	Nuclear factor κB
OPN	Osteopontin
PDGF	Platelet-derived growth factor
PTHrP	Parathyroid hormone-related protein
RANK	Receptor Activator of NFκB
RANKL	Receptor activator of NFκB ligand
SNO	Spindle-shaped N-cadherin+ osteoblast
SOST-1	Sclerostin 1
TGFβ	Transforming growth factor β
TSP-1	Thrombospondin-1
VCAM1	Vascular cell adhesion molecule-1

## References

[1] Coleman R.E. (2000). Management of bone metastases. Oncologist.

[2] Coleman R.E. (2001). Metastatic bone disease: Clinical features, pathophysiology and treatment strategies. Cancer Treat. Rev..

[3] Coleman R.E. (1997). Skeletal complications of malignancy. Cancer.

[4] Erler J.T., Bennewith K.L., Cox T.R., Lang G., Bird D., Koong A., Le Q.T., Giaccia A.J. (2009). Hypoxia-induced lysyl oxidase is a critical mediator of bone marrow cell recruitment to form the premetastatic niche. Cancer Cell.

[5] Croset M., Goehrig D., Frackowiak A., Bonnelye E., Ansieau S., Puisieux A., Clézardin P. (2014). TWIST1 expression in breast cancer cells facilitates bone metastasis formation. J. Bone Miner. Res..

[6] Sahay D., Leblanc R., Grunewald T.G.P., Ambatipudi S., Ribeiro J., Clézardin P., Peyruchaud O. (2015). The LPA1/ZEB1/miR-21-activation pathway regulates metastasis in basal breast cancer. Oncotarget.

[7] Lamouille S., Xu J., Derynck R. (2014). Molecular mechanisms of epithelial-mesenchymal transition. Nat. Rev. Mol. Cell Biol..

[8] Dave B., Mittal V., Tan N.M., Chang J.C. (2012). Epithelial-mesenchymal transition, cancer stem cells and treatment resistance. Breast Cancer Res..

[9] Gunasinghe N.P., Wells A., Thompson E.W., Hugo H.J. (2012). Mesenchymal-epithelial transition (MET) as a mechanism for metastatic colonisation in breast cancer. Cancer Metastasis Rev..

[10] Bendinelli P., Maroni P., Matteucci E., Desiderio M.A. (2015). HGF and TGFβ1 differently influenced Wwox regulatory function on Twist program for mesenchymal-epithelial transition in bone metastatic versus parental breast carcinoma cells. Mol. Cancer.

[11] Sanders J.L., Chattopadhyay N., Kifor O., Yamaguchi T., Butters R.R., Brown E.M. (2000). Extracellular calcium-sensing receptor expression and its potential role in regulating parathyroid hormone-related peptide secretion in human breast cancer cell lines. Endocrinology.

[12] Kim W., Takyar F.M., Swan K., Jeong J., VanHouten J., Sullivan C.A., Dann P., Yu H., Fiaschi-Taesch N., Chang W. (2016). Calcium-sensing receptor (CaSR) promotes breast cancer by stimulating intracrine actions of parathyroid hormone-related protein. Cancer Res..

[13] Fidler I.J. (2003). The pathogenesis of cancer metastasis: the “seed and soil” hypothesis revisited. Nat. Rev. Cancer.

[14] Kang Y., Siegel P.M., Shu W., Drobnjak M., Kakonen S.M., Cordón-Cardo C., Guise T.A., Massagué J. (2003). A multigenic program mediating breast cancer metastasis to bone. Cancer Cell.

[15] Müller A., Homey B., Soto H., Ge N., Catron D., Buchanan M.E., McClanahan T., Murphy E., Yuan W., Wagner S.N. (2001). Involvement of chemokine receptors in breast cancer metastasis. Nature.

[16] Ha H.K., Lee W., Park H.J., Lee S.D., Lee J.Z., Chung M.K. (2011). Clinical significance of CXCL16/CXCR6 expression in patients with prostate cancer. Mol. Med. Rep..

[17] Golan K., Kollet O., Lapidot T. (2013). Dynamic cross talk between S1P and CXCL12 regulates hematopoietic stem cells migration, development and bone remodeling. Pharm. Basel. Switz..

[18] Smith M.C.P., Luker K.E., Garbow J.R., Prior J.L., Jackson E., Piwnica-Worms D., Luker G.D. (2004). CXCR4 regulates growth of both primary and metastatic breast cancer. Cancer Res..

[19] Sun X., Cheng G., Hao M., Zheng J., Zhou X., Zhang J., Taichman R.S., Pienta K.J., Wang J. (2010). CXCL12/CXCR4/CXCR7 chemokine axis and cancer progression. Cancer Metastasis Rev..

[20] Richert M.M., Vaidya K.S., Mills C.N., Wong D., Korz W., Hurst D.R., Welch D.R. (2009). Inhibition of CXCR4 by CTCE-9908 inhibits breast cancer metastasis to lung and bone. Oncol. Rep..

[21] Hu W., Zhen X., Xiong B., Wang B., Zhang W., Zhou W. (2008). CXCR6 is expressed in human prostate cancer in vivo and is involved in the in vitro invasion of PC3 and LNCap cells. Cancer Sci..

[22] Saidak Z., Boudot C., Abdoune R., Petit L., Brazier M., Mentaverri R., Kamel S. (2009). Extracellular calcium promotes the migration of breast cancer cells through the activation of the calcium sensing receptor. Exp. Cell Res..

[23] Adams G.B., Chabner K.T., Alley I.R., Olson D.P., Szczepiorkowski Z.M., Poznansky M.C., Kos C.H., Pollak M.R., Brown E.M., Scadden D.T. (2006). Stem cell engraftment at the endosteal niche is specified by the calcium-sensing receptor. Nature.

[24] Mundy G.R. (2002). Metastasis to bone: causes, consequences and therapeutic opportunities. Nat. Rev. Cancer.

[25] Edwards J.R., Mundy G.R. (2011). Advances in osteoclast biology: Old findings and new insights from mouse models. Nat. Rev. Rheumatol..

[26] Santini D., Schiavon G., Vincenzi B., Gaeta L., Pantano F., Russo A., Ortega C., Porta C., Galluzzo S., Armento G. (2011). Receptor activator of NF-κB (RANK) expression in primary tumors associates with bone metastasis occurrence in breast cancer patients. PLoS ONE.

[27] Jones D.H., Nakashima T., Sanchez O.H., Kozieradzki I., Komarova S.V., Sarosi I., Morony S., Rubin E., Sarao R., Hojilla C.V. (2006). Regulation of cancer cell migration and bone metastasis by RANKL. Nature.

[28] Boyce B.F., Xing L. (2008). Functions of RANKL/RANK/OPG in bone modeling and remodeling. Arch Biochem. Biophys..

[29] Arrigoni C., de Luca P., Gilardi M., Previdi S., Broggini M., Moretti M. (2014). Direct but not indirect co-culture with osteogenically differentiated human bone marrow stromal cells increases RANKL/OPG ratio in human breast cancer cells generating bone metastases. Mol. Cancer..

[30] Ikeda T., Utsuyama M., Hirokawa K. (2001). Expression profiles of receptor activator of nuclear factor κB ligand, receptor activator of nuclear factor κB, and osteoprotegerin messenger RNA in aged and ovariectomized rat bones. J. Bone Miner Res..

[31] Nakashima T., Hayashi M., Fukunaga T., Kurata K., Oh-hora M., Feng J.Q., Bonewald L.F., Kodama T., Wutz A., Wagner E.F. (2011). Evidence for osteocyte regulation of bone homeostasis through RANKL expression. Nat. Med..

[32] Xiong J., Onal M., Jilka R.L., Weinstein R.S., Manolagas S.C., O’Brien C.A. (2011). Matrix-embedded cells control osteoclast formation. Nat. Med..

[33] Shiozawa Y., Havens A.M., Jung Y., Ziegler A.M., Pedersen E.A., Wang J., Wang J., Lu G., Roodman G.D., Loberg R.D. (2008). Annexin II/annexin II receptor axis regulates adhesion, migration, homing, and growth of prostate cancer. J. Cell Biochem..

[34] Martin T.A., Jiang W.G. (2009). Loss of tight junction barrier function and its role in cancer metastasis. Biochim. Biophys. Acta..

[35] Wang H., Yu C., Gao X., Welte T., Muscarella A.M., Tian L., Zhao H., Zhao Z., Du S., Tao J. (2015). The osteogenic niche promotes early-stage bone colonization of disseminated breast cancer cells. Cancer Cell..

[36] Clëzardin P. (2009). Integrins in bone metastasis formation and potential therapeutic implications. Curr. Cancer Drug Targets.

[37] Sung V., Stubbs J.T., Fisher L., Aaron A.D., Thompson E.W. (1998). Bone sialoprotein supports breast cancer cell adhesion proliferation and migration through differential usage of the αvβ3 and αvβ5 integrins. J. Cell Physiol..

[38] Zhao Y., Bachelier R., Treilleux I., Pujuguet P., Peyruchaud O., Baron R., Clément-Lacroix P., Clézardin P. (2007). Tumor αvβ3 integrin is a therapeutic target for breast cancer bone metastases. Cancer Res..

[39] Sloan E.K., Pouliot N., Stanley K.L., Chia J., Moseley J.M., Hards D.K., Anderson R.L. (2006). Tumor-specific expression of αvβ3 integrin promotes spontaneous metastasis of breast cancer to bone. Breast Cancer Res..

[40] Michigami T., Shimizu N., Williams P.J., Niewolna M., Dallas S.L., Mundy G.R., Yoneda T. (2000). Cell-cell contact between marrow stromal cells and myeloma cells via VCAM-1 and α4β1-integrin enhances production of osteoclast-stimulating activity. Blood.

[41] Malanchi I., Santamaria-Martínez A., Susanto E., Peng H., Lehr H.A., Delaloye J.F., Huelsken J. (2012). Interactions between cancer stem cells and their niche govern metastatic colonization. Nature.

[42] Zhang X.H.F., Wang Q., Gerald W., Hudis C.A., Norton L., Smid M., Foekens J.A., Massagué J. (2009). Latent bone metastasis in breast cancer tied to Src-dependent survival signals. Cancer Cell.

[43] Zhang X.H., Jin X., Malladi S., Zou Y., Wen Y.H., Brogi E., Smid M., Foekens J.A., Massagué J. (2013). Selection of bone metastasis seeds by mesenchymal signals in the primary tumor stroma. Cell.

[44] Winkler I.G., Barbier V., Nowlan B., Jacobsen R.N., Forristal C.E., Patton J.T., Magnani J.L., Lévesque J.P. (2012). Vascular niche E-selectin regulates hematopoietic stem cell dormancy, self renewal and chemoresistance. Nat. Med..

[45] Shiozawa Y., Pedersen E.A., Patel L.R., Ziegler A.M., Havens A.M., Jung Y., Wang J., Zalucha S., Loberg R.D., Pienta K.J. (2010). GAS6/AXL axis regulates prostate cancer invasion, proliferation, and survival in the bone marrow niche. Neoplasia.

[46] Li L., Bhatia R. (2011). Molecular pathways: Stem cell quiescence. Clin. Cancer Res..

[47] Lamorte S., Remédio L., Dias S. (2009). Communication between bone marrow niches in normal bone marrow function and during hemopathies progression. Hematol. Rev..

[48] Ghajar C.M., Peinado H., Mori H., Matei I.R., Evason K.J., Brazier H., Almeida D., Koller A., Hajjar K.A., Stainier D.Y. (2013). The perivascular niche regulates breast tumor dormancy. Nat. Cell. Biol..

[49] Kobayashi A., Okuda H., Xing F., Pandey P.R., Watabe M., Hirota S., Pai S.K., Liu W., Fukuda K., Chambers C. (2011). Bone morphogenetic protein 7 in dormancy and metastasis of prostate cancer stem-like cells in bone. J. Exp. Med..

[50] Sharma S., Xing F., Liu Y., Wu K., Said N., Pochampally R., Shiozawa Y., Lin H.K., Balaji K.C., Watabe K. (2016). Secreted protein acidic and rich in cysteine (SPARC) mediates metastatic dormancy of prostate cancer in the bone. J. Biol. Chem..

[51] Bragado P., Estrada Y., Parikh F., Krause S., Capobianco C., Farina H.G., Schewe D.M., Aguirre-Ghiso J.A. (2013). TGF-β2 dictates disseminated tumour cell fate in target organs through TGF-β-RIII and p38α/β signalling. Nat. Cell Biol..

[52] Lu X., Mu E., Wei Y., Riethdorf S., Yang Q., Yuan M., Yan J., Hua Y., Tiede B.J., Lu X. (2011). VCAM-1 promotes osteolytic expansion of indolent bone micrometastasis of breast cancer by engaging α4β1-positive osteoclast progenitors. Cancer Cell.

[53] Braun S., Vogl F.D., Naume B., Janni W., Osborne M.P., Coombes R.C., Schlimok G., Diel I.J., Gerber B., Gebauer G. (2005). A pooled analysis of bone marrow micrometastasis in breast cancer. N. Engl. J. Med..

[54] Loeb S., Feng Z., Ross A., Trock B.J., Humphreys E.B., Walsh P.C. (2011). Can we stop prostate specific antigen testing 10 years after radical prostatectomy?. J. Urol..

[55] Morrissey C., Roudier M.P., Dowell A., True L.D., Ketchanji M., Welty C., Corey E., Lange P.H., Higano C.S., Vessella R.L. (2013). Effects of androgen deprivation therapy and bisphosphonate treatment on bone in patients with metastatic castration-resistant prostate cancer: results from the University Of Washington Rapid Autopsy Series. J. Bone Miner Res..

[56] Lam H.M., Vessella R.L., Morrissey C. (2014). The role of the microenvironment-dormant prostate disseminated tumor cells in the bone marrow. Drug. Discov. Today.

[57] Mansi J.L., Gogas H., Bliss J.M., Gazet J.C., Berger U., Coombes R.C. (1999). Outcome of primary-breast-cancer patients with micrometastases: A long-term follow-up study. Lancet.

[58] Lilleby W., Stensvold A., Mills I.G., Nesland J.M. (2013). Disseminated tumor cells and their prognostic significance in nonmetastatic prostate cancer patients. Int. J. Cancer.

[59] Morgan T.M., Lange P.H., Porter M.P., Lin D.W., Ellis W.J., Gallaher I.S., Vessella R.L. (2009). Disseminated tumor cells in prostate cancer patients after radical prostatectomy and without evidence of disease predicts biochemical recurrence. Clin. Cancer Res..

[60] Coleman R.E., Gregory W., Marshall H., Wilson C., Holen I. (2013). The metastatic microenvironment of breast cancer: Clinical implications. Breast.

[61] Ehninger A., Trumpp A. (2011). The bone marrow stem cell niche grows up: Mesenchymal stem cells and macrophages move in. J. Exp. Med..

[62] Kusumbe A.P., Ramasamy S.K., Itkin T., Mäe M.A., Langen U.H., Betsholtz C., Lapidot T., Adams R.H. (2016). Age-dependent modulation of vascular niches for haematopoietic stem cells. Nature.

[63] Broxmeyer H.E., Orschell C.M., Clapp D.W., Hangoc G., Cooper S., Plett P.A., Liles W.C., Li X., Graham-Evans B., Campbell T.B. (2005). Rapid mobilization of murine and human hematopoietic stem and progenitor cells with AMD3100, a CXCR4 antagonist. J. Exp. Med..

[64] Haider M.T., Holen I., Dear T.N., Hunter K., Brown H.K. (2014). Modifying the osteoblastic niche with zoledronic acid in vivo—Potential implications for breast cancer bone metastasis. Bone.

[65] Shiozawa Y., Pedersen E.A., Havens A.M., Jung Y., Mishra A., Joseph J., Kim J.K., Patel L.R., Ying C., Ziegler A.M. (2011). Human prostate cancer metastases target the hematopoietic stem cell niche to establish footholds in mouse bone marrow. J. Clin. Investig..

[66] Wang N., Reeves K.J., Brown H.K., Fowles A.C., Docherty F.E., Ottewell P.D., Croucher P.I., Holen I., Eaton C.L. (2015). The frequency of osteolytic bone metastasis is determined by conditions of the soil, not the number of seeds; evidence from in vivo models of breast and prostate cancer. J. Exp. Clin. Cancer Res..

[67] Price T.T., Burness M.L., Sivan A., Warner M.J., Cheng R., Lee C.H., Olivere L., Comatas K., Magnani J., Lyerly H.K. (2016). Dormant breast cancer micrometastases reside in specific bone marrow niches that regulate their transit to and from bone. Sci. Transl. Med..

[68] Wang N., Docherty F.E., Brown H.K., Reeves K.J., Fowles A.C., Ottewell P.D., Dear T.N., Holen I., Croucher P.I., Eaton C.L. (2014). Prostate cancer cells preferentially home to osteoblast-rich areas in the early stages of bone metastasis: evidence from in vivo models. J. Bone Miner Res..

[69] Ghajar C.M. (2015). Metastasis prevention by targeting the dormant niche. Nat. Rev. Cancer.

[70] Croucher P.I., McDonald M.M., Martin T.J. (2016). Bone metastasis: The importance of the neighbourhood. Nat. Rev. Cancer.

[71] Sosa M.S., Bragado P., Aguirre-Ghiso J.A. (2014). Mechanisms of disseminated cancer cell dormancy: An awakening field. Nat. Rev. Cancer.

[72] Anthony B.A., Link D.C. (2014). Regulation of hematopoietic stem cells by bone marrow stromal cells. Trends Immunol..

[73] Mishra A., Wang J., Shiozawa Y., McGee S., Kim J., Jung Y., Joseph J., Berry J.E., Havens A., Pienta K.J. (2012). Hypoxia stabilizes GAS6/Axl signaling in metastatic prostate cancer. Mol. Cancer Res..

[74] Chen G., Deng C., Li Y.P. (2012). TGF-β and BMP signaling in osteoblast differentiation and bone formation. Int. J. Biol. Sci..

[75] Nguyen D.X., Bos P.D., Massagué J. (2009). Metastasis: From dissemination to organ-specific colonization. Nat. Rev. Cancer.

[76] Becker S., Becker-Pergola G., Wallwiener D., Solomayer E.F., Fehm T. (2006). Detection of cytokeratin-positive cells in the bone marrow of breast cancer patients undergoing adjuvant therapy. Breast Cancer Res. Treat..

[77] Wong C.W., Lee A., Shientag L., Yu J., Dong Y., Kao G., Al-Mehdi A.B., Bernhard E.J., Muschel R.J. (2001). Apoptosis: An early event in metastatic inefficiency. Cancer Res..

[78] Minn A.J., Kang Y., Serganova I., Gupta G.P., Giri D.D., Doubrovin M., Ponomarev V., Gerald W.L., Blasberg R., Massagué J. (2005). Distinct organ-specific metastatic potential of individual breast cancer cells and primary tumors. J. Clin. Investig..

[79] Massagué J., Obenauf A.C. (2016). Metastatic colonization by circulating tumour cells. Nature.

[80] Eck S.M., Côté A.L., Winkelman W.D., Brinckerhoff C.E. (2009). CXCR4 and matrix metalloproteinase-1 are elevated in breast carcinoma-associated fibroblasts and in normal mammary fibroblasts exposed to factors secreted by breast cancer cells. Mol. Cancer Res..

[81] Orimo A., Gupta P.B., Sgroi D.C., Arenzana-Seisdedos F., Delaunay T., Naeem R., Carey V.J., Richardson A.L., Weinberg R.A. (2005). Stromal fibroblasts present in invasive human breast carcinomas promote tumor growth and angiogenesis through elevated SDF-1/CXCL12 secretion. Cell.

[82] Prajapati P., Lambert D.W. (2016). Cancer-associated fibroblasts—Not-so-innocent bystanders in metastasis to bone?. J. Bone Oncol..

[83] Ottewell P.D., Wang N., Meek J., Fowles C.A., Croucher P.I., Eaton C.L., Holen I. (2014). Castration-induced bone loss triggers growth of disseminated prostate cancer cells in bone. Endocr. Relat. Cancer.

[84] Zheng Y., Zhou H., Fong-Yee C., Modzelewski J.R.K., Seibel M.J., Dunstan C.R. (2008). Bone resorption increases tumour growth in a mouse model of osteosclerotic breast cancer metastasis. Clin. Exp. Metastasis.

[85] Ottewell P.D., Wang N., Brown H.K., Reeves K.J., Fowles C.A., Croucher P.I., Eaton C.L., Holen I. (2014). Zoledronic acid has differential antitumor activity in the pre- and postmenopausal bone microenvironment in vivo. Clin. Cancer Res..

[86] Ottewell P.D., O’Donnell L., Holen I. (2015). Molecular alterations that drive breast cancer metastasis to bone. BoneKEy Rep..

[87] Croucher P.I., Parker B.S., Corcoran N., Rogers M.J. (2015). Bone turnover markers and prostate cancer: Not just a measure of bone disease?. Eur. Urol..

[88] Lipton A., Chapman J.A.W., Demers L., Shepherd L.E., Han L., Wilson C.F., Pritchard K.I., Leitzel K.E., Ali S.M., Pollak M. (2011). Elevated bone turnover predicts for bone metastasis in postmenopausal breast cancer: Results of NCIC CTG MA. 14. J. Clin. Oncol..

[89] Roodman G.D. (2005). High bone turnover markers predict poor outcome in patients with bone metastasis. J. Clin. Oncol..

[90] Anders C.K., Johnson R., Litton J., Phillips M., Bleyer A. (2009). Breast cancer before age 40 years. Semin. Oncol..

[91] Lin D.W., Porter M., Montgomery B. (2009). Treatment and survival outcomes in young men diagnosed with prostate cancer: A population-based cohort study. Cancer.

[92] Rakhra K., Bachireddy P., Zabuawala T., Zeiser R., Xu L., Kopelman A., Fan A.C., Yang Q., Braunstein L., Crosby E. (2010). CD4(+) T cells contribute to the remodeling of the microenvironment required for sustained tumor regression upon oncogene inactivation. Cancer Cell.

[93] Baschuk N., Rautela J., Parker B.S. (2015). Bone specific immunity and its impact on metastasis. BONEKey Rep..

[94] Le Gall C., Bellahcène A., Bonnelye E., Gasser J.A., Castronovo V., Green J., Zimmermann J., Clézardin P. (2007). A cathepsin K inhibitor reduces breast cancer induced osteolysis and skeletal tumor burden. Cancer Res..

[95] Bellahcène A., Bachelier R., Detry C., Lidereau R., Clézardin P., Castronovo V. (2007). Transcriptome analysis reveals an osteoblast-like phenotype for human osteotropic breast cancer cells. Breast Cancer Res. Treat..

[96] Pawelek J.M., Chakraborty A.K. (2008). Fusion of tumour cells with bone marrow-derived cells: A unifying explanation for metastasis. Nat. Rev. Cancer.

[97] Lawson M.A., McDonald M.M., Kovacic N., Khoo W.H., Terry R.L., Down J., Kaplan W., Paton-Hough J., Fellows C., Pettitt J.A. (2015). Osteoclasts control reactivation of dormant myeloma cells by remodelling the endosteal niche. Nat. Commun..

[98] Barkan D., El Touny L.H., Michalowski A.M., Smith J.A., Chu I., Davis A.S., Webster J.D., Hoover S., Simpson R.M., Gauldie J. (2010). Metastatic growth from dormant cells induced by a col-I-enriched fibrotic environment. Cancer Res..

[99] Aguirre-Ghiso J.A., Liu D., Mignatti A., Kovalski K., Ossowski L. (2001). Urokinase receptor and fibronectin regulate the ERKMAPK to p38MAPK activity ratios that determine carcinoma cell proliferation or dormancy in vivo. Mol. Biol. Cell.

[100] Christensen J., Shastri V.P. (2015). Matrix-metalloproteinase-9 is cleaved and activated by Cathepsin K. BMC Res. Notes.

[101] Kollet O., Dar A., Shivtiel S., Kalinkovich A., Lapid K., Sztainberg Y., Tesio M., Samstein R.M., Goichberg P., Spiegel A. (2006). Osteoclasts degrade endosteal components and promote mobilization of hematopoietic progenitor cells. Nat. Med..

[102] Swarbrick A., Roy E., Allen T., Bishop J.M. (2008). Id1 cooperates with oncogenic Ras to induce metastatic mammary carcinoma by subversion of the cellular senescence response. Proc. Natl. Acad. Sci. USA.

[103] Stankic M., Pavlovic S., Chin Y., Brogi E., Padua D., Norton L., Massagué J., Benezra R. (2013). TGF-β-Id1 signaling opposes Twist1 and promotes metastatic colonization via a mesenchymal-to-epithelial transition. Cell Rep..

[104] Brown H.K., Ottewell P.D., Evans C.A., Holen I. (2012). Location matters: Osteoblast and osteoclast distribution is modified by the presence and proximity to breast cancer cells in vivo. Clin. Exp. Metastasis.

[105] Buijs J.T., Stayrook K.R., Guise T.A. (2012). The role of TGF-β in bone metastasis: novel therapeutic perspectives. BoneKEy Rep..

[106] Le Pape F., Vargas G., Clézardin P. (2016). The role of osteoclasts in breast cancer bone metastasis. J. Bone Oncol..

[107] Croset M., Kan C., Clézardin P. (2015). Tumour-derived miRNAs and bone metastasis. BoneKEy Rep..

[108] Ell B., Mercatali L., Ibrahim T., Campbell N., Schwarzenbach H., Pantel K., Amadori D., Kang Y. (2013). Tumor-induced osteoclast miRNA changes as regulators and biomarkers of osteolytic bone metastasis. Cancer Cell.

[109] Sugatani T., Vacher J., Hruska K.A. (2011). A microRNA expression signature of osteoclastogenesis. Blood.

[110] Valencia K., Luis-Ravelo D., Bovy N., Antón I., Martínez-Canarias S., Zandueta C., Ormazábal C., Struman I., Tabruyn S., Rebmann V. (2014). miRNA cargo within exosome-like vesicle transfer influences metastatic bone colonization. Mol. Oncol..

[111] Weilbaecher K.N., Guise T.A., McCauley L.K. (2011). Cancer to bone: A fatal attraction. Nat. Rev. Cancer.

[112] Duong L.T., Wesolowski G.A., Leung P., Oballa R., Pickarski M. (2014). Efficacy of a cathepsin K inhibitor in a preclinical model for prevention and treatment of breast cancer bone metastasis. Mol. Cancer Ther..

[113] Taubenberger A.V. (2014). In vitro microenvironments to study breast cancer bone colonisation. Adv. Drug. Deliv. Rev..

[114] Rochefort G.Y. (2014). The osteocyte as a therapeutic target in the treatment of osteoporosis. Ther. Adv. Musculoskelet. Dis..

